# Development of targeted biologics for systemic lupus erythematosus

**DOI:** 10.1186/1479-5876-10-S2-A23

**Published:** 2012-10-17

**Authors:** Peter E Lipsky

**Affiliations:** 1 Charlottesville, Virginia, USA

## 

The prototypic autoimmune disease, systemic lupus erythematosus (SLE), is known to be associated with a number of genetic polymorphisms that can affect B cell function as well as polyclonal B cell hyperactivity. Developing an understanding of the complex nature of human B cell activation and differentiation has permitted an assessment of whether specific stages of B cell maturation are affected by the tendency for polyclonal B cell activation. Moreover, the analysis of perturbations of the specific stages of B cell maturation has generated new information on whether abnormalities in B cell differentiation are primarily involved in SLE immunopathology or, rather, are secondary to the inflammatory environment characteristic of subjects with this autoimmune disease. Multivariant flow cytometric analysis has documented abnormalities in B cell maturation that are primarily associated with lupus, or, alternatively related to disease duration, disease activity and concomitant medication. Together, these analyses have provided new insights on the role of B cell over-reactivity in SLE. Characterization of peripheral blood B cell subsets in patients with SLE has provided unique opportunities to identify abnormalities among pre-naïve, transitional, pre-naïve, memory B cells and in particular plasmablasts/plasma cells including indications for defects in negative selection of autoreactive B cells at certain stages. The pathogenic impact of all of these individual disturbances remains less clear, although the findings have served to identify SLE as associated with numerous abnormalities in B cell development. Moreover, active SLE is characterized by overactive T cell dependent germinal center-like reactions that produce expanded and largely unregulated numbers of memory B cells and plasma cells, thereby contributing to SLE pathogenesis. Finally, an understanding of the nature of the B cell abnormalities in SLE has generated new targets for therapy of this disease. The first of these, belimumab, a monoclonal antibody to the B cell survival factor B Lymphocyte stimulator (BLyS), has proven successful in treating patients with SLE. Additional approaches that target specific stages of B cell activation or differentiation are in development for treatment of SLE.

